# Metagenomic next-generation sequencing of bronchoalveolar lavage fluid assists in the diagnosis of pathogens associated with lower respiratory tract infections in children

**DOI:** 10.3389/fcimb.2023.1220943

**Published:** 2023-09-26

**Authors:** Yunjian Xu, Yueting Jiang, Yan Wang, Fanlin Meng, Wenyan Qin, Yongping Lin

**Affiliations:** ^1^ Department of Clinical Laboratory, The Key Laboratory of Advanced Interdisciplinary Studies Center, The First Affiliated Hospital of Guangzhou Medical University, National Center for Respiratory Medicine, National Clinical Research Center for Respiratory Disease, Guangzhou, China; ^2^ CapitalBio Technology Inc., Beijing, China; ^3^ Department of Laboratory Medicine, Cancer Hospital Chinese Academy of Medical Sciences, Shenzhen Center, Shenzhen, China

**Keywords:** lower respiratory tract infections, pathogen diagnosis, bronchoalveolar lavage fluid, metagenomic next-generation sequencing, traditional methods, medication adjustment, children

## Abstract

Worldwide, lower respiratory tract infections (LRTI) are an important cause of hospitalization in children. Due to the relative limitations of traditional pathogen detection methods, new detection methods are needed. The purpose of this study was to evaluate the value of metagenomic next-generation sequencing (mNGS) of bronchoalveolar lavage fluid (BALF) samples for diagnosing children with LRTI based on the interpretation of sequencing results. A total of 211 children with LRTI admitted to the First Affiliated Hospital of Guangzhou Medical University from May 2019 to December 2020 were enrolled. The diagnostic performance of mNGS versus traditional methods for detecting pathogens was compared. The positive rate for the BALF mNGS analysis reached 95.48% (95% confidence interval [CI] 92.39% to 98.57%), which was superior to the culture method (44.07%, 95% CI 36.68% to 51.45%). For the detection of specific pathogens, mNGS showed similar diagnostic performance to PCR and antigen detection, except for *Streptococcus pneumoniae*, for which mNGS performed better than antigen detection. *S. pneumoniae*, cytomegalovirus and *Candida albicans* were the most common bacterial, viral and fungal pathogens. Common infections in children with LRTI were bacterial, viral and mixed bacterial-viral infections. Immunocompromised children with LRTI were highly susceptible to mixed and fungal infections. The initial diagnosis was modified based on mNGS in 29.6% (37/125) of patients. Receiver operating characteristic (ROC) curve analysis was performed to predict the relationship between inflammation indicators and the type of pathogen infection. BALF mNGS improves the sensitivity of pathogen detection and provides guidance in clinical practice for diagnosing LRTI in children.

## 1 Introduction

Lower respiratory tract infections (LRTI) are the deadliest infectious diseases worldwide and the fourth leading cause of death (World Health Organization, 2020). Early and accurate identification of the etiology of LRTI is essential for effective pathogen targeted therapy. However, pathogen identification is limited due to the limitations of traditional microbiological detection. Diagnosis is further complicated by noninfectious inflammatory syndromes that mimic LRTI (Langelier et al., 2018a). Currently, traditional detection methods, including microscopy, pathogen culture and isolation, biochemical testing, immunology, and polymerase chain reaction (PCR) testing, are used mainly to identify LRTI pathogens in children. However, these methods have shortcomings in terms of sensitivity, specificity, timeliness, and amount of information obtained (Jain et al., 2015; Zhang et al., 2018; Rajapaksha et al., 2019). Moreover, it is impossible to quickly identify unknown or rare pathogenic microorganisms. In the absence of a clear microbiological diagnosis, clinicians may assume that symptoms are caused by noninfectious inflammation and prescribe empirical corticosteroids, which can exacerbate occult infections (Wilson et al., 2014).

Metagenomic next-generation sequencing (mNGS) is a novel technique for rapid, efficient, and unbiased acquisition of nucleic acid sequence information that can be used to identify pathogens in a given sample. Moreover, the advent of rapid mNGS has extended its applications from laboratory research to clinical diagnostics (Goldberg et al., 2015). mNGS has been used mainly to diagnose emerging pathogens and rare infectious diseases (Yao et al., 2016). For example, in the unusual pneumonia outbreak reported in Wuhan in December 2019, mNGS was performed using RNA extracted from patient bronchoalveolar lavage fluid (BALF), which rapidly identified a novel coronavirus pathogen (SARS-CoV-2) present in high abundance (Chen et al., 2020). Rapid and accurate diagnostic methods for detecting pathogenic microorganisms are extremely crucial for disease control and treatment. mNGS is already being successfully applied for pneumonia, meningitis, liver abscess, endometritis, and endophthalmitis diagnosis using BALF, sputum, cerebrospinal fluid, pleural fluid, and vitreous humor specimens (Ai et al., 2018; Langelier et al., 2018b; Moreno et al., 2018; Miller et al., 2019).

In recent years, the feasibility of mNGS for etiological detection and identification of respiratory tract infections has been demonstrated (Miao et al., 2018; Li et al., 2020). However, numerous challenges remain, including the interpretation of mNGS results, human genome interference and other common issues. In addition, there are few studies on BALF mNGS in a large cohort of children with LRTI, which needs to be addressed. Here, we summarize the mNGS results for 229 BALF samples from 211 children with LRTI and sought to validate the value of mNGS for diagnosing children with LRTI based on the interpretation of sequencing results.

## 2 Materials and methods

### 2.1 Patients

This study retrospectively analyzed the medical records of 211 children with LRTI who were admitted to the First Affiliated Hospital of Guangzhou Medical University from May 2019 to December 2020. The study was approved by the institutional ethics committee of the First Affiliated Hospital of Guangzhou Medical University (No. 2021K-40). The medical records contained patient information, clinical diagnosis and symptoms, results of mNGS and traditional microbiological assays, information on relevant clinical laboratory tests and clinical medication information. The inclusion criteria were as follows: 1) chest imaging revealing abnormalities; 2) infection symptoms that did not improve after empirical treatment; patients presenting with persistent expectoration, fever, and shortness of breath, and patients with unsatisfactory clinical outcomes; re-examination of chest imaging showed no improvement, and the etiology needed to be clarified; and 3) for immunocompromised patients complicated with septic shock or multiple organ failure, the attending physician may have advised the use of mNGS. The exclusion criteria were patients unable to fulfill the required medical follow-up. In this study, 229 BALF samples from 211 children with LRTI were assessed using mNGS (DNA and RNA) assays. Resampling for testing occurred in the following circumstances: 1) results were not consistent with diagnosis or treatment; 2) infection improved but was not cured; 3) samples were resampled for mNGS detection in cases with negative mNGS results but clinical manifestations of infection; and 4) repeated infection was observed for a long time.

A total of 39 patients were considered immunocompromised when clinically diagnosed, including 1) patients with blood-related diseases (e.g., aplastic anemia, thalassemia, and congenital neutrophil deficiency) or 2) cancers (e.g., solid malignancy and hematological malignancy), 3) patients who received invasive surgery (e.g., heart surgery and a patient with tumor resection), and 4) patients diagnosed with kidney diseases (e.g., uremia and nephrotic syndrome) or 5) multiple organ failure and other autoimmune deficiencies (Ramírez, 2013; Tecklenborg et al., 2018; Spoor et al., 2019; Zinter et al., 2019; El Hasbani et al., 2022; Xi et al., 2022; Zaimoku et al., 2022).

### 2.2 Flow of BALF sample collection

BALF specimen collection was performed as previously described with some modifications (Collins et al., 2014; Hogea et al., 2020). 1) Site selection: Lesion segments were selected for patients with limited lesions. For diffuse lesions, the right middle lobe of the lung or the lingual segment of the left upper lobe of the lung was selected. 2) Local anesthesia: 1-2 mL of 2% lidocaine was injected into the biopsy hole of the lavage lung segment to perform local anesthesia; 1-2 mL of 2% lidocaine could also be administered to patients under intravenous combination anesthesia who had strong airway reactions. 3) Saline injection: After the tip of the bronchoscope was wedged in the opening of the target bronchial segment or inserted subterminal, sterile saline (37°C or room temperature) was rapidly injected through the operating channel in a total volume of 20-30 mL, and multiple injections (3-10 mL each time) were performed. 4) Negative pressure suction: Immediately after the injection of saline, BALF was obtained by suction with an appropriate negative pressure (commonly recommended below 100-200 mm Hg), and the total recovery rate was more than 40%. 5) BALF collection: The recovered fluid contained approximately 10 mL of secretions from bronchial terminals and alveoli. The potentially contaminated portion from the front was discarded, and the remaining portion of at least 3 mL was collected for immediate inspection.

### 2.3 Traditional methods detection

Simultaneously, sputum, throat swab and blood samples were collected. All the above samples, including BALF samples, were immediately subjected to the following laboratory tests. Culture: Blood agar, Sabouraud dextrose agar, chocolate agar, and MacConkey agar used to manually inoculate and culture pathogens were purchased from Autobio Diagnostics Co., Ltd. (China). Bacteria were cultured at 35°C for 24 to 48 hours, and fungi were cultured for 7 days. The VITEK® 2 system (France) was used for automated bacterial and fungal identification. The sample types included BALF, sputum, blood, pleural effusion, cerebrospinal fluid, urine, or stool samples. Nucleic acid detection: The processes were carried out according to the protocols of commercial kits. In this study, 15 nucleic acid detection kits were used from seven suppliers; the detection kits were for *Mycobacterium tuberculosis* (MTB) and *Chlamydia trachomatis* (Qiagen, Shenzhen, China), cytomegalovirus (CMV), Epstein−Barr virus (EBV), enterovirus, coxsackievirus, human herpes simplex virus 1 (HSV1), and *Mycoplasma pneumoniae* (MP) (Daan Gene, China), adenovirus (HAdV) (Hecin, China), respiratory syncytial virus (RSV) and influenza virus A/B (Huayin, China), HSV2 (Biot Gene, China), hepatitis B virus (Amplly, China), and hepatitis C virus (Sansure, China). The sample types included BALF, sputum, swab, blood, pleural effusion, cerebrospinal fluid, urine, or stool samples. Antibody detection: In this study, the MP antibody detection test kit (passive agglutination method) was purchased from Fujirebio (Japan). This kit only detects MP antibodies (IgM and IgG) and does not directly detect MP. Therefore, a positive result does not confirm MP infection, and comprehensive evaluation of the patient’s condition should be made by combining clinical symptoms and test results. The sample type was blood. Antigen detection: The processes were carried out according to the protocols of the commercial kits. In this study, five antigen detection kits were used from four suppliers; the detection kits were for *S. pneumoniae* (colloidal gold method) (Abbott, USA), rotavirus (colloidal gold method) (Wantai, China), *Aspergillus* spp. (ELISA), *Cryptococcus* (ELISA) (Genobio, China), and seven respiratory viruses (namely, influenza A/B virus, human parainfluenza virus 1-3 (HPIV 1-3), RSV, HAdV) (immunofluorescence method) (B&C, China). The sample types for detecting *S. pneumoniae* were cerebrospinal fluid and urine, those for detecting *Aspergillus* spp. or *Cryptococcus* were BALF and blood, those for detecting rotavirus were stool, and those for detecting the seven respiratory viruses were nasal swabs. The commercial kits used in this study were all certified and approved for clinical testing by the China National Medical Products Administration.

Quality control of the manual culture method: The quality control strains were *Pseudomonas aeruginosa* ATCC 27853, *Escherichia coli* ATCC 25922, *Escherichia coli* ATCC 35218, *Staphylococcus aureus* ATCC 25923, and *Staphylococcus aureus* ATCC 29213. Quality control of automated culture and identification: conducted every two weeks. The quality control strains were *Staphylococcus sciuri* ATCC 29061, *Candida albicans* ATCC 14053, *Eikenella corrodens* BAA-1152, *Enterobacter aerogenes* ATCC 13048 and *Enterobacter hormaechei* ATCC 700323. Positive controls for Hepatitis B/C virus nucleic acid testing were obtained from Conchestan (China). Controls are included in the remaining commercial kits used for clinical testing.

### 2.4 Metagenomic next-generation sequencing using BALF samples

BALF samples were obtained; 3 mL of BALF was placed in a sterile sputum container, stored at 4°C, and sent to CapitalBio (Guangzhou, China) for mNGS detection. DNA from each BALF sample (0.5 mL) was extracted using a QIAamp DNA Microbiome Kit (Cat#51704, QIAGEN, Germany), and RNA was extracted using a QIAamp Viral RNA Mini Kit (Cat#52904, QIAGEN, Germany). The extracted RNA was reverse transcribed using random primers, and cDNA was pooled with DNA from the same sample for sequencing library preparation. The pooled nucleic acid was enzymatically fragmented to a size of 200-300 bp, and sequencing libraries were constructed through end repair, adapter ligation and PCR amplification. Sequencing templates were prepared with OneTouch2 System (Life Technologies, USA), and after quality control, sequencing was performed using a BioelectronSeq 4000 sequencer (CapitalBio, China) based on a semiconductor platform Ion Torrent Proton™ sequencer. A negative control sample consisting of water was used in each run to monitor potential contamination.

The original sequencing data were subjected to quality control, and adapter reads, low quality, reads with N (represents uncertain base information) > 5, reads with lengths less than 50 bp, or low complexity were removed. The remaining high-quality sequencing data were mapped to the human reference genome grch38 for depletion of human host sequences using Bowtie2 software. Subsequently, nonhuman sequences were classified by simultaneous alignment to the genomic sequence databases downloaded from the NCBI and PATRIC, which contain 13,992 bacterial species, 1,659 fungal species, 13,000 viral species and 287 parasitic pathogens. To judge the suspected pathogens in the clinical samples, we reviewed data for different types of samples from healthy people and calculated relevant reference values, including the hit read number and coverage of all bacteria, fungi, viruses and parasites detected. Moreover, pathogens detected in the negative control sample (water) were removed from the results for the clinical samples. The final pathogen detection results included a list of suspected pathogens, the number of hit reads and genome-level coverage statistics.

Human nucleic acid depletion is one of the challenges of mNGS (Diao et al., 2021). In this study, the following two methods were mainly used to remove human nucleic acids. 1) The processed BALF sample was centrifuged, and pathogens were distributed in the supernatant and precipitate based on their different structures. The precipitate contains the majority of human host cells and pathogens with cellular structures such as bacteria and fungi. Differential lysis was performed based on the different structures of human host cells and pathogen cells, and the nucleic acids released from human host cells were digested using nucleases for the first depletion of host nucleic acids. This process was repeated once. 2) The supernatant containing viral nucleic acids and a small portion of human host cells was subjected to two rounds of centrifugation to remove as many human host cells as possible. Then, the pathogen sequences were enriched. After filtering and deduplication, the average total number of reads was 9,518,921 (320,578-26,524,467). Human reads accounted for an average of 69.96% (6.56%-93.69%); microorganism reads accounted for an average of 6.70% (0.12%-66.81%); and unmapped reads accounted for an average of 23.34% (6.06%-60.30%).

mNGS for each BALF cost approximately $600, and the result could be obtained about 24 hours of the sample’s arrival at the testing laboratory.

### 2.5 Criteria for a positive mNGS result

1) Positive indicators of comprehensive interpretation were as follows: microbial characteristics (cell wall thickness, genome size), number of detected sequences, genome coverage percent and estimated concentration (copies/mL). Above the threshold of the parameter, the organism was judged as a high- or medium-confidence pathogen. 2) Additional positive indicators were as follows: bacteria, fungi, or parasites: cover length > 3000 bp; viruses: cover length > 300 bp. 3) When clinically confirming pathogenic microorganisms, a comprehensive judgment was made based on the pathogen, sample, and clinical characteristics.

### 2.6 Statistical analysis

The sensitivity (TPR), specificity (TNR), positive predictive value (PPV), and negative predictive value (NPV) were calculated and compared between mNGS and traditional pathogen detection methods. Statistical analyses were performed using SPSS software v.24.0. Pearson’s chi-square test or Fisher’s exact test was used for discrete variables where appropriate. P values < 0.05 were considered significant, and all tests were two-tailed.

## 3 Results

### 3.1 Samples and patient characteristics

A total of 211 children with LRTI were the subjects of this study (**Supplementary Table 1**). There were 121 males and 90 females. Their average age was 5.00 ± 4.30 years. Among the 211 patients, 172 were non-immunocompromised, and 39 were immunocompromised (**Table 1**). Among the 39 immunocompromised children with LRTI were diagnosed with nine kinds of diseases, namely, aplastic anemia, thalassemia, congenital neutrophil deficiency, invasive surgery, solid malignancy, kidney disease, multiple organ failure, hematological malignancy, and other autoimmune deficiencies (**Table 1**; **Supplementary Table 1**).

**Table 1:** Characteristics of 211 children with LRTI.

Characteristic	mNGS (n = 211)
Age (years)	5.00 ± 4.30
Sex	
Female, n (%)	90 (42.65%)
Male, n (%)	121 (57.35%)
Non-immunocompromised, n (%)	172 (81.52%)
Immunocompromised, n (%)	39 (18.48%)
Aplastic anemia	2 (5.13%)
Thalassemia	3 (7.69%)
Congenital neutrophil deficiency	1 (2.56%)
Hematological malignancy	19 (48.72%)
After invasive surgery	2 (5.13%)
Solid malignancy	3 (7.69%)
Kidney disease	2 (5.13%)
Multiple organ failure	1 (2.56%)
Other autoimmune deficiency	6 (15.38%)

### 3.2 Comparison of diagnostic performances between mNGS and traditional methods in non-immunocompromised children with LRTI

**Supplementary Tables 2**, **3** list the etiological detection results for 172 enrolled non-immunocompromised children with LRTI, including the results for 186 samples by mNGS, 177 samples by culture, 165 samples by PCR detection, 136 samples by antibody detection (serological assay) for MP, 137 samples by antigen detection for bacteria and fungi, and 154 samples by antigen detection for viruses. The etiological detection results for the non-immunocompromised children with LRTI showed 176 cases to be positive when using BALF for mNGS, with a positive rate of 94.62% (176/186, 95% CI 91.35% to 97.90%). Seventy-eight cases were positive based on the culture method, with a positive rate of 44.07% (78/177, 95% CI 36.68% to 51.45%). The culture method is regarded as the gold standard for the clinical diagnosis of bacterial and fungal infections. Comparing the culture method with the corresponding mNGS results, the TPR, TNR, PPV and NPV of mNGS were 98.72%, 7.07%, 45.56% and 87.50%, respectively. A total of 95.48% (169/177, 95% CI 92.39% to 98.57%) of the BALF mNGS results were positive, which was superior to the culture method (χ^2^= 110.92, P <.001) (**Supplementary Table 3**). Antibody detection (serological assay) is the main method for the clinical diagnosis of MP. The positive rate of the serological assay for MP was 57.35% (78/136, 95% CI 48.93% to 65.77%). The corresponding BALF mNGS positive rate was 7.35% (10/136, 95% CI 2.91% to 11.80%), which was lower than that for serological detection (χ^2^= 77.68, P <.001). The concordance rate between mNGS and serological detection for MP was 47.06%, while the concordance rate between mNGS and PCR was 81.73%, indicating that there may be a high false-positive rate for serological detection of MP (**Table 2**, **Supplementary Table 3**). PCR and antigen detection were used for clinically suspected specific pathogens. Most of the concordance rates of mNGS and the traditional methods were above 85%. The positive rates of mNGS and traditional methods (PCR, antigen detection for viruses) for specific pathogens differed, but there were no significant differences (**Table 2**). Antigen detection of *Aspergillus* spp. and *Cryptococcus*, did not differ significantly from mNGS, while for *S. pneumoniae*, the positive rate of BALF mNGS was 40.91% (36/88, 95% CI 30.43% to 51.39%), which was superior to the rate for antigen detection of *S. pneumoniae* (7.95%, 7/88, 95% CI 2.19% to 13.72%) (χ^2^= 25.88, P <.001) (**Table 2**).

**Table 2:** Diagnostic performance of mNGS compared with traditional methods for non-immunocompromised children with LRTI.

	Pathogen	Sample number	Corresponding mNGS positive	Traditional method positive	Double positive	Concordance rate (%)	P value
**PCR**	MTB	142	1	2	1	99.30	1
	MP	104	12	19	6	81.73	0.242
	*Chlamydia trachomatis*	23	1	0	0	95.65	1
	CMV	65	12	16	8	81.54	0.393
	HAdV	79	16	20	12	84.81	0.448
	RSV	8	2	1	1	87.5	1
	Influenza A virus	21	1	2	1	95.24	1
	Influenza B virus	20	0	0	0	100.00	/
	EBV	30	0	4	0	86.67	0.112
	Enterovirus	8	0	0	0	100.00	/
	Coxsackie virus	3	0	0	0	100.00	/
	Hepatitis B virus	1	0	1	0	0.00	1
	Hepatitis C virus	2	0	0	0	100.00	/
	HSV 1	2	0	0	0	100.00	/
	HSV 2	2	0	0	0	100.00	/
**Detection of antibody (serological assay)**	MP	136	10	78	8	47.06	**< 0.001**
**Detection of bacterial and fungal antigens**	*S. pneumoniae*	88	36	7	6	64.77	**< 0.001**
	*Aspergillus* spp.	67	4	0	0	94.03	0.119
	*Cryptococcus*	68	0	1	0	98.53	0.181
**Detection of viral antigens**	Influenza A virus	153	2	1	1	99.35	1
	Influenza B virus	153	0	0	0	100.00	/
	HPIV 1	153	2	2	0	97.39	1
	HPIV 2	153	0	0	0	100.00	/
	HPIV 3	153	11	3	3	94.77	0.052
	RSV	153	11	11	8	96.08	1
	HAdV	153	18	8	7	92.16	0.063
	Rotavirus	14	0	0	0	100.00	/

MTB, Mycobacterium tuberculosis; MP, Mycoplasma pneumoniae; CMV, cytomegalovirus; HAdV, adenovirus; EBV, Epstein-Barr virus; RSV, respiratory syncytial virus; RhV, rhinovirus; HPIV, human parainfluenza virus; HSV, herpes simplex virus. “Concordance rate” represents the total of the positive and negative concordance rates.

The bold represent significant differences in data and can be replaced with regular fonts in this table.

The results showed no significant differences in the detection of pathogens between mNGS and PCR or in antigen detection, except for *S. pneumoniae*. In addition, BALF mNGS was superior to the culture method and antigen detection for *S. pneumoniae*, but the positive rate for diagnosing MP was lower than that of the serological assay.

### 3.3 Comparison of pathogens detected by the culture method and mNGS in non-immunocompromised children with LRTI

**Figure 1:**
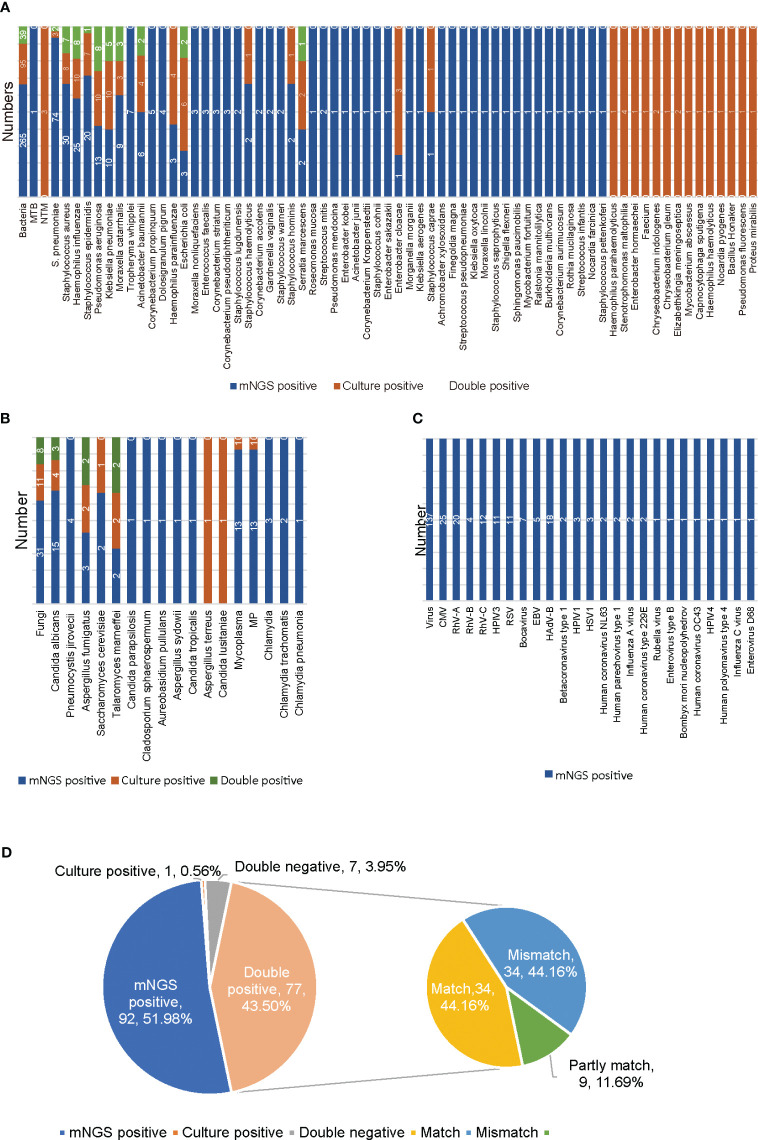
Results of mNGS and culture for non-immunocompromised children with LRTI. **(A)** The distribution of bacterial infections in 177 non-immunocompromised LRTI samples detected by mNGS and culture. **(B)** The distribution of fungal, *Mycoplasma* and *Chlamydia* infections in 177 non-immunocompromised LRTI samples detected by mNGS and culture. **(C)** The distribution of viral infections in 177 non-immunocompromised LRTI samples detected by mNGS. **(D)** The concordance between mNGS and culture for 177 non-immunocompromised LRTI samples. Culture results were used as the standard. Match indicates that all the pathogens detected by culture were also found by mNGS. Mismatch indicates that all the microorganisms detected by culture were not found by mNGS. Partly match indicates that some of the pathogens detected by culture were found by mNGS. MTB, *Mycobacterium tuberculosis*; NTM, nontuberculous mycobacteria; MP, *Mycoplasma pneumoniae*; CMV, cytomegalovirus; RhV, rhinovirus; HAdV, adenovirus; EBV, Epstein-Barr virus; RSV, respiratory syncytial virus; HPIV, human parainfluenza virus; HSV, herpes simplex virus.

The culture method is the primary method used in clinical microbiology laboratories for identifying bacterial and fungal pathogens of LRTI. Comparison of the results for mNGS and culture for the identification of suspected pathogens in all 177 samples is shown in **Figure 1**. mNGS and culture were both positive for 77 of the 177 samples (43.50%). Seven were both negative (3.95%), 92 samples were positive only by mNGS (51.98%), and only one sample was positive by culture (0.56%). For the 77 double-positive samples, the concordance between mNGS and culture was assessed as match (34, 44.16%), partly match (9, 11.69%), and mismatch (34, 44.16%) (**Figure 1D**). For culture results, 36 kinds of pathogens were detected, namely, 30 kinds of bacteria and six kinds of fungi (**Figures 1A, B**). The most frequently detected bacteria were *Pseudomonas aeruginosa* (10/177), *Klebsiella pneumoniae* (10/177) and *Haemophilus influenzae* (10/177), followed by *Staphylococcus aureus* (8/177) (**Figure 1A**). *Candida albicans* (4/177) was the most frequently detected fungus (**Figure 1B**). According to mNGS, 90 kinds of microorganisms were determined, namely, 24 kinds of viruses, 53 kinds of bacteria, 10 kinds of fungi, one *Mycoplasma* and two kinds of *Chlamydia* (**Figure 1**). Among the microbes detected, *S. pneumoniae* (74/177) was the most frequently detected bacterium, followed by *Staphylococcus aureus* (30/177), *Haemophilus influenzae* (25/177), *Staphylococcus epidermidis* (20/177) and *Pseudomonas aeruginosa* (13/177) (**Figure 1A**). The top six detected viruses were CMV (25/177), rhinovirus subtype A (RhV-A) (20/177), HAdV-B (18/177), RhV-C (12/177), human parainfluenza virus type 3 (HPIV3) and RSV (11/177) (**Figure 1C**). *Candida albicans* (15/177) was the most frequently detected fungus (**Figure 1B**). Among all BALF samples, only one case of MTB was detected (**Figure 1A**). A total of 38 species of bacteria and six species of fungi were detected only by mNGS. Fifteen kinds of bacteria and two kinds of fungi were detected only by culture. mNGS has a broader spectrum for pathogen detection and can detect more definite or probable pathogens than culture.

### 3.4 Comparison of types of pathogens detected in non-immunocompromised and immunocompromised children with LRTI

**Figure 2:**
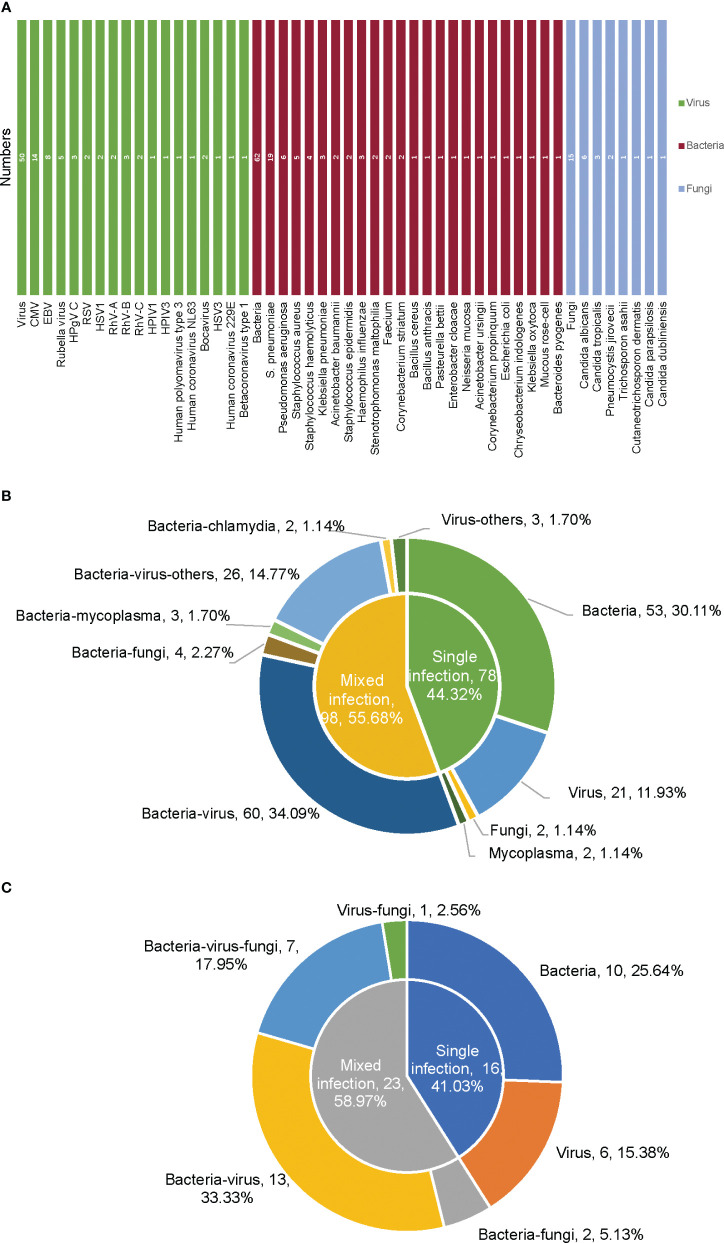
Types of pathogens based on mNGS in non-immunocompromised and immunocompromised children with LRTI. **(A)** Pathogen spectrum of infection in 39 immunocompromised children with LRTI detected by mNGS. **(B)** Pathogen types of infection in 172 non-immunocompromised children with LRTI detected by mNGS. **(C)** Pathogen types of infection in 39 immunocompromised children with LRTI detected by mNGS. CMV, cytomegalovirus; EBV, Epstein−Barr virus; RSV, respiratory syncytial virus; RhV, rhinovirus; HPIV, human parainfluenza virus; HSV, herpes simplex virus.

According to the mNGS results, the most frequently detected pathogens were bacteria, followed by viruses and fungi (**Figures 1A–C**, **2A**). For the 186 samples from 172 non-immunocompromised children, except for 10 samples for which no pathogens were detected, 44.32% of the infected children were diagnosed with single bacterial, viral, fungal, or *Mycoplasma* infection (30.11%, 53/176; 11.93%, 21/176; 1.14%, 2/176; 1.14%, 2/176). The mixed infections observed were mainly bacterial-viral (34.09%, 60/176) and bacterial-viral-fungal (10.23%, 18/176) (**Figures 1**, **2B**). For the 43 samples from 39 immunocompromised children, except for 4 samples for which no microbes were detected, 41.03% of the children were diagnosed with single bacterial or viral infections (25.64%, 10/39; 15.38%, 6/39). Mixed infections were mainly bacterial-viral (33.33%, 13/39) and bacterial-viral-fungal (17.95%, 7/39) (**Figure 2C**). Compared with the non-immunocompromised children with LRTI, the immunocompromised children with LRTI were more susceptible to mixed infections (58.97% > 55.68%, P = 0.708) and fungal infections (25.64% > 17.05%, P = 0.212); however, there were no significant differences.

### 3.5 Medication strategy adjustment according to mNGS detection

Complete information on medication orders was available for 125 of the 211 patients (**Supplementary Table 4**). Forty-six patients underwent medication adjustments within three days of obtaining the mNGS results. Thirty-two patients had their medication adjusted within 24 hours based on the mNGS results, and in five patients, their medication was adjusted within two days of obtaining the mNGS results. For the 37 patients (29.60%, 37/125) whose medications were adjusted based on the mNGS results within two days, the adjustments were as follows: antibacterial drugs were increased for 13 patients; antibacterial drugs were changed for 10; antifungal drugs were increased for six; antifungal drugs were reduced for two; antiviral drugs were increased for three; the antibacterial drug was replaced and the antifungal drug increased for one, the antiviral drug was reduced and the antibacterial drug replaced for one; and the antibacterial drug was reduced and the antifungal drug increased for one. 63.20% (79/125) of cases were abandoned for adjustment due to consistent results between previous antibiotic usage and mNGS testing. All 125 patients with mNGS test and complete medication information on medication orders were improved and discharged.

### 3.6 Inflammation indicators predict types of pathogen infection in children with LRTI

**Figure 3:**
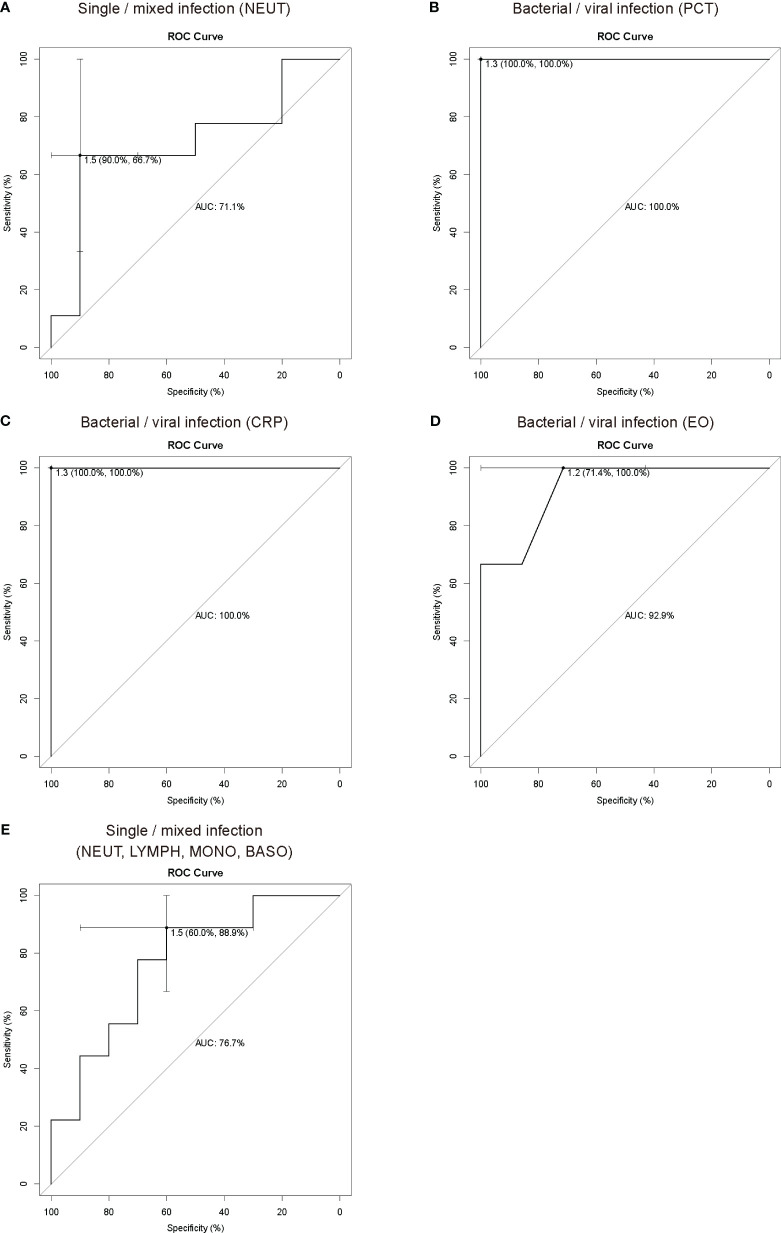
ROC curve analysis of inflammation indicators and pathogen types. ROC curve analysis showed that NEUT **(A)** could be used as an indicator to distinguish single infections from mixed infections, and PCT **(B)**, CRP **(C)** and EO **(D)** could be used as indicators to distinguish bacterial infections and viral infections. **(E)** ROC curve analysis showed that NEUT, LYMPH, MONO and BASO could be used in combination with multiple inflammation indicators to distinguish single infections from mixed infections. In ROC analysis, the sample size for the training set was fifty-nine, and the sample size for the test set was nineteen for distinguishing single (bacterial or viral) and mixed (bacterial-viral) infections. The sample size for the training set was thirty, and the sample size for the test set was ten for distinguishing single bacterial and viral infections. PCT, procalcitonin; CRP, C-reactive protein; NEUT, neutrophils; LYMPH, lymphocyte; MONO, monocyte; EO, eosinophils; BASO, basophil.

Receiver operating characteristic (ROC) curve analysis was performed to assess the predictive performance of inflammation indicators and the type of pathogen infection and to calculate the area under curve (AUC). In this study, 78 samples, namely, 28 bacterial, 12 viral, and 38 bacterial-viral infection samples with complete procalcitonin (PCT), C-reactive protein (CRP) and routine blood data, were used for analysis of inflammation indicators and types of infectious pathogens (**Supplementary Table 5**). Univariate logistic regression analysis showed that neutrophils (NEUT) were able to distinguish single (bacterial or viral) and mixed (bacterial and viral) infections, and the AUC value was 0.711 (**Figure 3A**). PCT, CRP and eosinophils (EO) were also able to distinguish bacterial infections from viral infections, with AUC values of 1, 1 and 0.929, respectively (**Figures 3B-D**). Multivariate logistic regression analysis showed that four indicators, NEUT, lymphocyte (LYMPH), monocyte (MONO) and basophil (BASO), were able to distinguish single infections from mixed infections (**Figure 3E**), with an AUC value of 0.767. However, there were no significant differences in the logistic regression analysis, and more samples from children with LRTI are needed to further study the relationship between inflammatory indicators and different types of infection.

## 4 Discussion

For LRTI, early etiological diagnosis is necessary. However, traditional culture methods are time-consuming and have low positive detection rates. mNGS is suitable for detecting pathogens that cannot be identified by other detection technologies and for patients who do not respond to standard antibacterial treatments (Li et al., 2020). For rare and slow-growing pathogenic microorganisms, mNGS has considerable advantages, such as reducing the time required for diagnosis and confirmation of mixed infections, facilitating targeted antibacterial therapy, and improving patient prognosis.

We know that the lung is not sterile and supports the existence of a different microbiota in the upper and lower compartments (Cabrera-Rubio et al., 2012). Given its operability, several noninvasive and invasive procedures have been used to surrogate or proxy lung tissue for sampling of the pulmonary environment (Yi et al., 2022). BALF, a common method for sampling the lung microbiome, is more similar to the lower airway than sputum, and has limited contamination from the upper airway or oral cavity. While other microbial sampling methods include bronchial brushing and tracheal aspirate, the application range is limited (Yi et al., 2022). BALF specimen collection in children should be carried out according to the corresponding standard requirements, but different medical institutions have their own procedures for BAL operations. During the collection of BALF samples, it is not possible to establish a personalized sample collection procedure based on the type of unknown microbe that causes pediatric lung infections. For this study, BALF sample collection was adapted from literature and the Guidelines for Specimen Collection, Transportation and Detection of Microorganisms in Respiratory Infections in Chinese Children and modified according to clinical practice. Approximately 20-30 mL of lavage fluid was used in this study, with a recovery volume of approximately 10 mL.

Among 229 BALF samples from 211 children with LRTI, 215 (93.89%) tested positive by mNGS. Thirteen patients underwent resampling tests. No. 4 underwent five mNGS tests, and No. 100 underwent four mNGS tests. The first four tests of No. 4 detected *S. pneumoniae*, and the last test did not detect it. mNGS testing can also indirectly reflect the treatment effect. No. 100 was an immunocompromised child who tested positive for rubella virus in all four tests, CMV and *S. pneumoniae* in three tests, and *Pseudomonas aeruginosa* in two tests. Although the pathogens were detected, the patient eventually died of X-linked immunodeficiency combined with severe pneumonia. Additionally, 11 patients underwent two mNGS tests. One patient tested negative in the first test but tested positive for *Enterococcus faecalis* and *Staphylococcus epidermidis* in the second test. One patient had completely different pathogen results in the two tests, while the remaining nine patients had partial overlap in the detected pathogens. mNGS resampling could quantitatively reflect the dynamic changes in pathogens and the treatment effect in patients. For 37 of the 125 patients who underwent mNGS, medication adjustments were performed to facilitate the optimization of clinical interventions based on the mNGS results. This suggests that the clinical value of mNGS using BALF in children with LRTI is substantial. Indeed, the mNGS detection method was superior to the culture method and was the same as the PCR identification method (**Table 2**), proving its effectiveness and accuracy in our research (Miao et al., 2018; Chen et al., 2021). BALF mNGS for MP had a lower positive rate than serological assays. There are three reasons for this result. One reason is that mNGS does not perform as well as serological assays for MP detection. Another reason is that MP might cause infection in other systems of the patient, such as the central nervous system, cardiovascular system, hematopoietic system, kidneys, or gastrointestinal system, and is not present in the LRTI samples (Narita, 2016; Al Busaidi et al., 2017). The third reason is that the high false-positive rate for MP in serological assays may affect the comparison of diagnostic performance between the two methods. The antibodies detected by MP serology in this study were IgM and IgG and could have indicated a past MP infection. On the other hand, for a reliable diagnosis of MP infection, paired sera, that is, acute and convalescent phase sera, are used to demonstrate a 4-fold titer increase or decrease. However, convalescent serum is difficult to obtain from children (Youn et al., 2010). In previous reports, a single titer ≥ 1:160 or 1:640 was considered to indicate acute MP infection (Matas et al., 1998; Kim et al., 2007). In our study, a single titer ≥ 1:40 was considered to indicate MP infection, possibly resulting in a high false-positive rate.

In this study, *S. pneumoniae*, CMV and *Candida albicans* were the most common bacterium, virus and fungus in children with LRTI, as in a previous report (Yang et al., 2022). However, the complete pathogen spectrum in our study differed from the study by Yang et al. Analyze the possible reason, our study utilized mNGS for DNA and RNA codetection, which can better assess the diagnostic value of mNGS in identifying RNA viruses causing LRTI, such as RSV, influenza virus, HPIV, and coronavirus. Compared to previous reports, the pathogen spectrum differs in children and adults or among different severity levels of disease (Huang et al., 2020; Li et al., 2020; Tsitsiklis et al., 2022; Xi et al., 2022). For hospitals with limited testing capabilities, empirical therapy based on local epidemiological characteristics detected by mNGS is recommended.

LRTI encompass a large and heterogeneous group of infections caused by bacteria, viruses, fungi and other etiologies. Inflammation indicators, such as CRP, PCT, and the white blood cell (WBC) count, are quantifiable, commonly available, and reflect underlying biological processes as well as disease severity, and their combination can characterize some specific infections. For example, PCT and CRP have been proven useful for helping to differentiate between pure SARS-CoV-2 or secondary bacterial infection and guiding the use of antibiotic therapy (Pink et al., 2021), and the PCT, CRP and WBC count can be combined as effective indicators for the identification of acute bacterial or nonbacterial infections in children (Li et al., 2021). Thus, the combination of multiple inflammation indicators has crucial clinical value. mNGS is high throughput and unbiased and simultaneously identifies bacterial, fungal, viral, parasitic, atypical and novel pathogens (Gu et al., 2019). In contrast, traditional culture methods can only identify culturable bacteria and fungi. Although PCR and antigen detection are rapid and accurate for pathogen identification, it requires prior knowledge or assumption of the pathogen type, and the detection throughput is relatively limited (Qian et al., 2020; Diao et al., 2021). Therefore, only mNGS can completely distinguish different types of pathogen infections. According to the results of mNGS in this study, the common infections in children with LRTI were bacterial, viral, and mixed bacterial-viral infections. In this study, univariate and multivariate logistic regression were used to assess the relationship between inflammation indicators and the types of infectious pathogens. NEUT were used to distinguish single and mixed infections; PCT, CRP or EO were used to distinguish bacterial and viral infections. Multivariate logistic regression analysis showed that NEUT, LYMPH, MONO and BASO could be used to distinguish single infections from mixed infections. Despite no significant difference, these measures are feasible and have important clinical value. The lack of significance might be due to an insufficient sample size. PCT or CRP has a high AUC value for distinguishing bacterial and viral infections, consistent with previous reports (Simon et al., 2004), but the inadequate sample size contributed to the exceptionally high AUC values. More samples from children with LRTI are needed to further investigate the relationship between inflammatory indicators and different types of infection.

mNGS is changing the way physicians diagnose and treat infectious diseases due to its wide range of applications, including assessing antimicrobial resistance, the microbiome, human host gene expression and oncology (Chiu and Miller, 2019). Empirical use of broad-spectrum antibiotics early in the course of treatment results in false-negatives when using traditional detection methods, although mNGS is less affected by antibiotic use (Miao et al., 2018; Diao et al., 2021). mNGS may reveal a massive amount of clinically irrelevant pathogens in the diagnosis of pulmonary infections, but it may improve the diagnostic yield, which might actually benefit clinical decision-making (Qian et al., 2020). Clinicians should combine traditional tests, clinical manifestations, immune status, underlying diseases and antibiotic use to determine the clinical significance of the microbe. In a subset of patients with underlying diseases or low immunity, colonization may lead to LRTI (Langelier et al., 2018b; Diao et al., 2021). Although mNGS is considered a promising experimental technique, there are several barriers to overcome, such as depletion of human nucleic acids, discrimination between colonization and infection, and high cost, before large scale clinical application (Diao et al., 2021).

This study is not without limitations. The BALF mNGS results were not confirmed by PCR or other traditional methods; thus, the results for microorganisms detected by mNGS should be combined with epidemiological and clinical characteristics before a pathogenic microbe can be identified. In addition, the sample size was insufficient to evaluate the relationship between the combination of inflammation indicators and the types of infectious pathogens based on the results of mNGS. For mNGS, there is no uniform standard for modifying or guiding clinical treatment strategies (Li et al., 2020). Finally, we suggest that mNGS may have high sensitivity for identifying early pathogens for which detection is usually time-consuming. With proper patient selection, sample processing and data interpretation, mNGS is expected to be a promising technique for the diagnosis and tailored treatment of clinical infectious diseases.

In conclusion, mNGS was found to be effective for pathogen diagnosis and informative for medication adjustment in children with LRTI in this retrospective study. Although mNGS has limitations, it has advantages compared with traditional methods. More pathogens can be detected using BALF mNGS, and it is also suitable for use as a supplementary method.

## Data availability statement

The data presented in the study are deposited in the FigShare repository, accession number: 10.6084/m9.figshare.24164913.

## Ethics statement

The studies involving humans were approved by Medical Ethics Committee of the First Affiliated Hospital of Guangzhou Medical University. The studies were conducted in accordance with the local legislation and institutional requirements. The ethics committee/institutional review board waived the requirement of written informed consent for participation from the participants or the participants’ legal guardians/next of kin because this study was an anonymous retrospective analysis, the ethics committee approved the application for waiver of informed consent.

## Author contributions

All authors have materially participated in this study and manuscript preparation. YX and YJ carried out all the molecular genetic analyses, participated in the design of the work and wrote the manuscript. YW and FM collected the clinical data, analyzed the data, and participated in conceiving the work. YL and WQ designed the work, drafted, and revised the manuscript. All authors contributed to the article and approved the submitted version.

## Supplementary material

Supplementary Table 1 Clinical information for 211 children with LRTI.

Supplementary Table 2 The mNGS sequencing data of 229 samples.

Supplementary Table 3 Results of pathogenic microorganisms’ detection in non-immunocompromised children with LRTI.

Supplementary Table 4 Medication information before and after mNGS detection in children with LRTI.

Supplementary Table 5 Inflammation indicator data.
